# Simultaneous Monitoring of the Evolution of Chemical Parameters in the Fermentation Process of Pineapple Fruit Wine Using the Liquid Probe for Near-Infrared Coupled with Chemometrics

**DOI:** 10.3390/foods11030377

**Published:** 2022-01-28

**Authors:** Sumaporn Kasemsumran, Antika Boondaeng, Kraireuk Ngowsuwan, Sunee Jungtheerapanich, Waraporn Apiwatanapiwat, Phornphimon Janchai, Jiraporn Meelaksana, Pilanee Vaithanomsat

**Affiliations:** 1 Laboratory of Non-Destructive Quality Evaluation of Commodities, Kasetsart Agricultural and Agro-Industrial Product Improvement Institute (KAPI), Kasetsart University, Bangkok 10900, Thailand; aapkrn@ku.ac.th (K.N.); aapsnj@ku.ac.th (S.J.); 2 Laboratory of Enzyme and Microbiology, KAPI, Kasetsart University, Bangkok 10900, Thailand; aapakb@ku.ac.th (A.B.); aapwpa@ku.ac.th (W.A.); aappmj@ku.ac.th (P.J.); aapjom@ku.ac.th (J.M.); aappln@ku.ac.th (P.V.)

**Keywords:** near-infrared spectroscopy, searching combination moving window partial least squares, pineapple, fruit wine, fermentation, liquid probe

## Abstract

This study used Fourier transform-near-infrared (FT-NIR) spectroscopy equipped with the liquid probe in combination with an efficient wavelength selection method named searching combination moving window partial least squares (SCMWPLS) for the determination of ethanol, total soluble solids, total acidity, and total volatile acid contents in pineapple fruit wine fermentation using *Saccharomyces cerevisiae* var. *burgundy*. Two fermentation batches were produced, and the NIR spectral data of the calibration samples in the wavenumber range of 11,536–3952 cm^−1^ were obtained over ten days of the fermentation period. SCMWPLS coupled with second derivatives searched and optimized spectral intervals containing useful information for building calibration models of four parameters. All models were validated by test samples obtained from an independent fermentation batch. The SCMWPLS models showed better predictions (the lowest value of prediction error and the highest value of residual predictive deviation) with acceptable statistical results (under confidence limits) among the results achieved by using the whole region. The results of this study demonstrated that FT-NIR spectroscopy using a liquid probe coupled with SCMWPLS could select the optimized wavelength regions while reducing spectral points and increasing accuracy for simultaneously monitoring the evolution of four chemical parameters in pineapple fruit wine fermentation.

## 1. Introduction

Pineapple (*Ananas comosus* L.) originates from South America and is one of the most favoured subtropical fruits cultivated (above 20% of the tropical fruit generated in the world) and consumed worldwide. It is a drought-tolerant plant with good taste [1]. The top three pineapple producers worldwide in 2019 were reported to be Costa Rica (3328.10 × 10^3^ metric tons), the Philippines (2747.86 × 10^3^ metric tons), and Brazil (2426.53 × 10^3^ metric tons), while Thailand is ranked seventh (1679.67 × 10^3^ metric tons) [2]. The fruit is frequently consumed fresh and used in the food industry (canned fruit, jam, and concentrated juice) for alcoholic beverage production [3] and fibre production [1]. In Thailand, pineapple wine is popularly consumed because of its unique taste, colour, and flavour. The consumption trend of wine made from pineapple and other fruits is likely to increase, especially among health-conscious consumers, because fruit wines are also nutritious and healthy [4,5].

Wine is an undistilled alcoholic beverage obtained from grapes or other fruits and plants, sugar, innate microorganisms, and yeast in suitable proportions [5,6]. Some sweet wine usually takes about ten days or more for the fermentation process before complete sugar conversion [7]. Wine composition is mainly composed of water and ethanol (97% *w*/*w* of the constituents in wine), which defines wine properties together with the viscosity, polarity, and solubility [8,9]. The other components (3% *w*/*w* of the constituents in wine) of phenolic compounds, sugars, glycerol, proteins, amino acids, organic acids, and inorganic compounds deliver the perceived colour, aroma, and flavour of wine, including sensory attributes (astringency and bitterness) that affect the quality and consumer preference [10]. Fermentation is an essential step in the production process owing to various physicochemical changes that occur during this step [11]. The wine industry requires analytical tools to rapidly determine components of fruit juices and wines for significant decision making during fermentation. These tools must ensure speed, simplicity, low or no sample preparation and destruction, and unused reagents or solvents [12]. Gas chromatography or high-performance liquid chromatography is broadly used for intensive quality analysis of wine. However, these techniques are time-consuming, laborious, and complicated methods [13]. In contrast, vibrational spectroscopy techniques have increased widespread acceptance and utilisation because they are non-destructive, fast, and suitable for routine analysis [14]. NIR spectroscopy is a vibrational spectroscopic technique, which is a promising technique for multicomponent analysis in wine fermentation. The NIR region is associated with the overtone and combination bands of the fundamental molecular vibrations of the OH, CH, and NH functional groups [15,16,17] Therefore, most components show absorption in the NIR region. However, it was found that studies using infrared (IR) spectroscopy to monitor wine fermentation techniques were more widely reported than NIR techniques [11,18], since NIR absorption bands are far weaker than IR absorption bands and a number of bands overlap each other due to overtone and combination modes [15,16,17]. Especially for highly complex samples such as fermented fruit wine, many NIR bands overlap each other in their NIR spectra.

In order to extract useful information from such complex NIR spectra of fermented fruit wine samples, chemometric methods are employed for spectral analysis. The proposed NIR quantitative determination of the multiple components for monitoring the wine fermentation process is shown in Table 1. In the previously published reports, the classical partial least squares (PLS) method was generally employed in the applications (Table 1). Several spectral preprocessings, such as baseline correction [19], first derivative (FD) [19,20,21,22,23], second derivative (SD) [20,21,23], detrending (DT) [19], eliminating the constant offset (ECO) [20], min–max normalization (MMN) [20,21], multiplicative scattering correction (MSC) [19,20,21,22,23]. Savitzky–Golay (SG) smoothing [19], standard normal variate (SNV) [19], stepwise regression analysis (SRA) [22], straight-line subtraction (SLS) [21], minus a straight line (MSL) [20], support vector machine (SVM) [19], and vector normalization (VN) [20,21] were applied. There was also a report for the use of wavelength selection methods, namely regression coefficient analysis (RCA)-PLS, the successive projection algorithm (SPA)-PLS, interval (i)-PLS, and the genetic algorithm (GA)-PLS with the model developments, in the prediction of ethanol and total acid contents during Chinese rice wine fermentation. The prediction result of using the wavelength selection methods of RCA-SVM-PLS (RMSEP for ethanol = 2.60 g L^−1^) and GA-SVM-PLS (RMSEP for total acid = 0.10 g L^−1^) provided better predictive performance than using the full-spectrum SVM-PLS (RMSEP for ethanol = 4.94 g L^−1^ and for total acid = 0.14 g L^−1^) [19].

Accordingly, the wavelength selection method is a crucial tool for searching the relevant information to improve the quality of prediction models for NIR analysis in wine fermentation. Advanced chemometrics, namely searching combination moving window partial least squares (SCMWPLS) [24], has been proposed to improve the performance of a PLS model. It functions as a spectral selection method to locate and optimize informative regions through spectra. The ability of calibration models can be improved by building the PLS models using the optimized informative regions found by SCMWPLS. The potentials of SCMWPLS were demonstrated and appeared in previously published reports [24,25,26,27]. However, no reports appear to have been published on the quantification of multiple components in pineapple fruit wine during the fermentation process using NIR spectroscopy in combination with the wavelength selection method. Therefore, the objectives of this study were (1) to investigate the feasibility of using NIR spectroscopy coupled with SCMWPLS in finding and optimizing informative spectral regions for simultaneous monitoring of the evolution of ethanol, total soluble solids (TSS), total acidity (TA), and total volatile acids (TVA) in pineapple fruit wine during fermentation, and (2) the use of an NIR liquid probe for immediate monitoring without sample preparation.

## 2. Materials and Methods

### 2.1. Pineapple Wine

#### 2.1.1. Yeast Culture Preparation

*Saccharomyces cerevisiae* var. *burgundy*, the primary yeast for general wine fermentation used in this study, was obtained from the Institute of Food Research and Product Development (IFRPD), Kasetsart University, Thailand. Yeast strains were activated on YPD agar for 24 to 48 h before use. An inoculum of 5% (V V^−1^) was prepared by mixing pineapple juice with yeast colonies (1 × 10^5^ CFU mL^−1^) for an incubation time of 24 h as a starter.

#### 2.1.2. Preparation of Pineapple Must

Pineapple samples at the ripe stage were purchased from a local market in Bangkok, Thailand. They were cleaned, peeled, and crushed. The ratio of pineapple juice to water was adjusted to obtain a 2:1 optimum ratio. The initial sugar concentration of the pineapple juice was adjusted to 25 °Brix by adding sucrose. Potassium metabisulfite (K_2_S_2_O_5_) was then added for decontamination to achieve a 75–100 mg L^−1^ final concentration.

#### 2.1.3. Pineapple Juice Fermentation

The fermentation of pineapple wine was performed in a polyethylene terephthalate bottle with a working volume of 15 L. Inoculum yeast cultures (5% V V^−1^) were used as a starter for wine fermentation in sterile pineapple juice. Fermentation was conducted for ten days at a controlled temperature of 30 °C using a water bath system. In total, three batches of fermentations were independently performed and employed two batches for calibration development and one batch for testing the predictive performance of the calibration model in this study.

### 2.2. NIR Liquid Probe Spectral Acquisition

**Figure 1:**
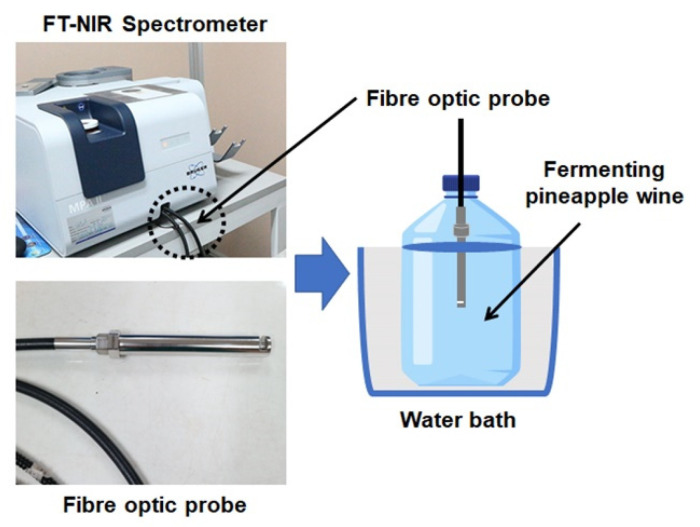
The scheme of the NIR measurement through the fermented bottle using the liquid fibre-optic probe.

A liquid fibre-optic probe (IN271P-02, Bruker Optics GmbH & Co. KG, Ettlingen, Germany) was used to collect the spectral data of the liquid wine sample in transflection mode. The NIR spectral information obtained using a transflectance probe provided an adequate signal dominating from both transmittance and reflectance information. The probe length was 14 cm, with a fixed optical path length of 2 mm (slit 1 mm). It consisted of fibre bundles with seven fibres in the stainless-steel probe housing with a sapphire window and an immersion probe designed for bubble shedding that is suitable for lab and process applications. The liquid probe was connected to an FT-NIR spectrophotometer (MPA II, Multi-Purpose Analyser, Bruker Optics GmbH & Co. KG, Ettlingen, Germany) for spectral acquisition between 11,536 and 3952 cm^−1^, and it was immersed in liquid samples for spectra acquisition. The air spectra were collected as the background for the measurements. For the establishment of the calibration model, a 30–40 mL sample was collected aseptically at the beginning of fermentation (0 h) and continued with a loop time of 3, 6, and 18 h every day until 240 h, for NIR scanning and chemical analysis. Before the sampling, the fermented samples in the bottle were randomly stirred by a sterile plastic rod and pipetted into a 50 mL sterile plastic tube. Each sample was divided for the NIR analysis and chemical analysis. To obtain the NIR information of a sample close to the actual samples as in the fermented bottle, all samples were directly scanned by the liquid probe without further preparation and the sterile plastic tube was used as the holder for the liquid sample. Regarding the acquisition process, the sample variation and light scattering variation were included in this study. After sampling, NIR measurements of the samples were immediately performed at a spectral resolution of 16 cm^−1^ with an interval of 8 cm^−1^ and a repeating 32-time scan per one measurement. Therefore, the data of 99 NIR spectral samples were obtained from one batch of the pineapple wine fermentation process (1 batch × 11 times (0 h and ten days) × 3 sampling times × 3 subsamples). The validation of model performance in NIR analysis was performed using the same process as described earlier, except the NIR spectral acquisition of samples that were measured by immersing the liquid fibre-optic probe in the fermented pineapple wine bottle. Furthermore, the plastic tube was used to cover the fibre line to avoid error from moving while the spectra were collected. The sample temperature was controlled at 30 °C throughout the experiment. Figure 1 shows the setting of the liquid fibre-optic probe for the NIR measurement of test samples during the fermentation process.

### 2.3. Pineapple Wine Chemical Analysis for Ethanol, Total Soluble Solids, Total Acidity, and Total Volatile Acids Using the Conventional Reference Methods

Four parameters of ethanol, total soluble solids (TSS), total acidity (TA), and total volatile acids (TVA) contents were monitored during fermentation processing and employed as the reference chemical data for NIR model development. For the chemical analysis, the samples were filtrated through the filter paper (No.1, Whatman) before determination as follows: (1) Ethanol concentration was assessed using gas chromatography (Chromosorb-103, GC4000; GL Sciences; Tokyo, Japan) with an HP5 capillary (30 m × 0.32 mm × 0.25 μm; JW Scientific; Folsom, CA, USA). (2) The TSS content in the sample was determined using a digital refractometer (PAL-1, ATAGO, Tokyo, Japan). (3) TA [28] and (4) TVA [29] were determined as citric acid and acetic acid, respectively, by titration using phenolphthalein as an indicator. For TA analysis, a sample (10 mL) was pipetted into a 250 mL Erlenmeyer flask containing 100 mL of hot distilled water. Phenolphthalein (2–3 drops) was added to the flask and titrated with 0.1 N NaOH until a pink colour appeared. TVA is separated from the wine samples by steam distillation before titration using sodium hydroxide (0.1 N) to obtain the pink end point indicated by the phenolphthalein solution. All measurements were performed in triplicate.

### 2.4. NIR Data Analysis

#### 2.4.1. Preprocessing Method

The NIR spectral data were collected using OPUS software (version 8.2: MPA II system, Bruker Optics GmbH & Co. KG, Ettlingen, Germany) and converted into JCAMP files. After that, the JCAMP files were imported into Unscrambler software (version 9.8: CAMO AS, Trondheim, Norway) and were independently performed without the method of spectral pretreatment (original spectral data) and with the method of second derivatives (SD) based on the Savitzky–Golay model (polynomial order = 2, number of smoothing points = 7) in order to remove the signal variation (spectral offsets and slopes) from light scattering in the fermented samples [15].

#### 2.4.2. Searching Combination Moving Window Partial Least Squares (SCMWPLS) Analysis

Two algorithms, moving window partial least squares regression (MWPLSR) [30] and SCMWPLS [24], were employed, respectively, in the calculation procedure. The calculation process of SCMWPLS is described as follows.

##### Step 1: MWPLSR Calculation

In MWPLSR, the calculation starts by building a series of PLS models in a spectral window ***X****_i_* (*m* × *h* matrix) that starts at the *i*th spectral channel and ends at the (*i* + *H* − 1)th spectral channel, which moves over the whole spectral region (*m* × *n* matrix). The spectra obtained in the spectral window is a sub-matrix ***X***_*i*_ (*n* × *h* matrix) containing the *i*th to the (*i* + *H* − 1)th columns of the calibration matrix ***X***. The PLS-1 models with different numbers of LV can then be built to relate the spectra in the window to the concentrations of the analyte as follows:

(1) $$y_{i}=X_{i}b_{i,k}+e_{i,k}$$

where *b*_*i*,*k*_ (*H* × 1 vector) is the regression coefficients vector estimated using PLS with *k*- LV and *e*_*i*,*k*_ is the residue vector obtained with a *k*-LV model. In this study, the window size for MWPLSR and the maximum LVs number were set to 20 spectral points and 10 LVs. The mean centred spectra in the whole region of 11,536–3952 cm^−1^ were applied. To avoid the effect on the residue lines obtained, the window size should be larger than the desired model dimensionality (LVs) and smaller than the spectral regions to be discovered. The window is moved over the whole spectral region. At each position, PLS models with varying LV numbers are built for the calibration samples, and the log of sums of squared residues (log(SSR)) are calculated with these PLS models and plotted as a function of the window position.

(2) $${\mathrm{SSR}}_{i}={\left(y_{i}-X_{i}{\hat{b}}_{i}\right)}^{t}(y_{i}-X_{i}{\hat{b}}_{i})$$

This will yield a number of residue lines, with each line associated with the log(SSR) for a certain LV in the corresponding window position. Then, the informative NIR spectral regions were discovered by plotting the residue lines corresponding to 1 to 10 LVs for PLS as a function of the position of the spectral window. A figure containing such residual lines provides information about informative spectral regions where residual lines show low values of SSR.

##### Step 2: SCMWPLS Calculation

After the selection of informative regions by MWPLSR, SCMWPLS starts to work for a given informative region with p spectral points by changing the moving window size *w* from 1 to *p*. A moving window is moved from the first spectral point to the (*p* − *w* + 1)th point over the informative region to collect all possible sub-windows for every window size. When *w* = 1, moving the window from the first to the end point will collect all possible sub-windows with the window size of one. Similarly, in other cases of *w*, all sub-windows with the size of *w* may be obtained. Therefore, this algorithm considers all possible spectral intervals in the range of the informative region. For every window, a PLS model with a selected LVs number is constructed, and the root mean square error of the calibration (RMSEC) is calculated. Comparing values of RMSEC for all sub-regions, the sub-region with the smallest value of RMSEC is considered the optimized informative region.

In this study, more than one informative region is suggested by MWPLSR, and the optimized combination of informative regions was performed by using the optimized sub-region as the base region. Next, SCMWPLS is performed for the second informative region, in which one uses the combinations of the base region and one of the possible spectral intervals selected from the second informative region to build PLS models and calculate their RMSEC values. After that, a new base region will be selected, which shows the smallest value of RMSEC. This calculation procedure is repeated to look for another new base region for the next informative region, until the last informative region is reached. After finishing calculations for all informative regions, the final base region is considered as the optimized combination. In SCMWPLS, the maximum LVs number is constrained and the LVs number selected by the validation method must not be larger than the maximum. The LVs number of the PLS model for an informative region can easily be estimated by regressing the spectra in the region against the concentrations. The LVs number is determined to be the number where the root mean square error of calibration (RMSEC) begins to decrease insignificantly with the increase in the LVs number. This LVs number is considered the maximum LVs number. All these calculations were carried out using in-house written programs in the MATLAB software (version 2020b: The MathWorks Inc., Natick, MA, USA).

#### 2.4.3. Calibration Development

PLS-1 (Unscrambler software) was applied to the spectral regions to develop the calibration models for the quantitative determination of ethanol, TSS, TA, and TVA in samples, simultaneously. The saturated NIR spectral region of 5248–4984 cm^−1^ was not included in the model developments as this spectral range is beyond the linear response region of the detector [19,25]. Two and one fermentation batches for pineapple wine samples were employed as the calibration set (*n* =198) and test set (*n* = 99), respectively. The full cross-validation method was used to find the optimum number of LVs for PLS by considering the number at which the lowest root mean squares error of cross-validation was obtained, and it increased from the next number [24,30,31]. The performances of the established calibration equations were further validated using the test set. To investigate the benefit of SCMWPLS, the PLS prediction results for the calibration models developed by using the spectral regions found by SCMWPLS were compared with those by using the full spectral regions according to the general PLS method.

### 2.5. Evaluation of the Predictive Ability of PLS and SCMWPLS Models

The prediction ability of models built by the whole NIR spectral region and the informative NIR region found by SCMWPLS were investigated and compared on the test set using the coefficient of determination (*R*^2^), root mean square error of calibration (RMSEC), root mean square error of prediction (RMSEP), and residual predictive deviation (RPD). An acceptable NIR model should present high values of *R*^2^ and RPD and low values of RMSEC and RMSEP. In addition, the accuracy of the best model was evaluated using values of the bias confidence limits (*T_b_*) and the unexplained error confidence limits (*T*_UE_), following the guidelines for the application of the NIR spectrometer described in ISO 12099 (2017) [32]. This verification method can assess the accepted model performance when the given standard error of prediction (SEP) and bias values fall within the confidence limits. Several earlier reports employed the standard ISO method, which has been detailed previously [33,34]. The statistics employed in this study are defined in Table 2.

## 3. Results and Discussion

### 3.1. Measured Chemical Characteristics of Pineapple Wines during Fermentation by Reference Methods

**Figure 2:**
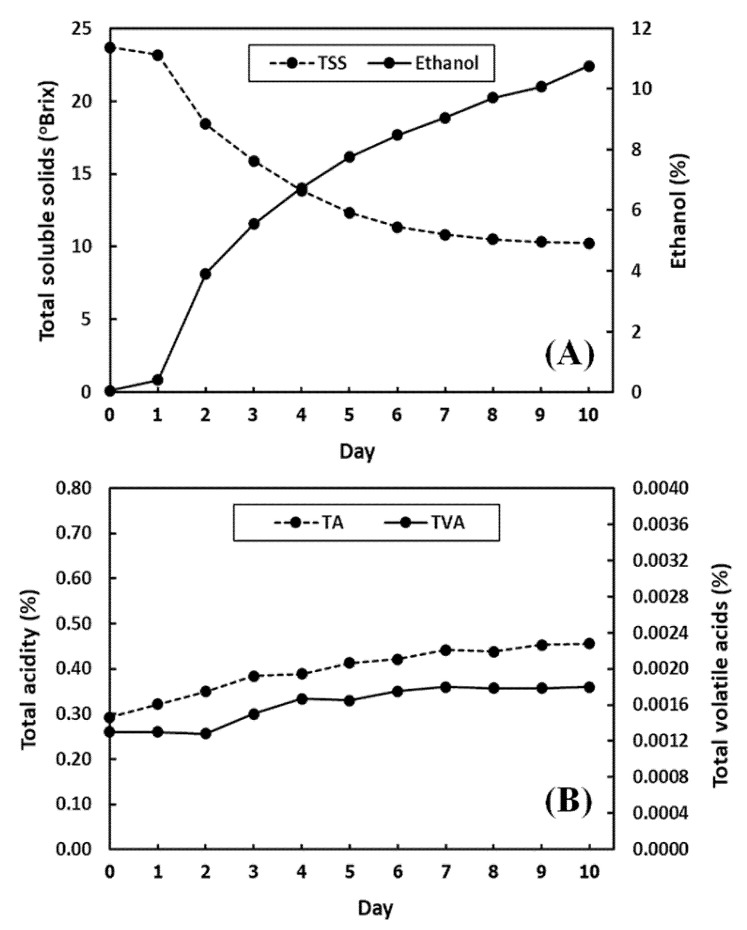
Monitoring of ethanol and total soluble solids (TSS) contents (**A**), and total acidity (TA) and total soluble solids (TVA) contents (**B**), for samples during the pineapple wine fermentation by the reference methods.

The results of the chemical analysis of pineapple wine samples during the process of fermentation are listed in Table 3. The results in a row show the averages of multiple measurements from two sample batches collected in the same day of fermentation. During the fermentation, the samples have an ethanol content of 0.0590 to 10.7592%, TTS in the range of 23.70 to 10.25 °Brix, TA of 0.2925 to 0.4558%, and TVA of 0.0013 to 0.0018%. The concentrations of ethanol, TA, and TVA in the samples increased with days of fermentation. On the other hand, the concentration of TTS in the sample decreased. Among the four analysts, ethanol and TSS values have higher variation than TA and TVA values as shown in Figure 2. As a result, the amount of TSS decreases rapidly because *Saccharomyces cerevisiae* var. *burgundy* produces the invertase enzyme that breaks down sucrose into glucose and fructose [35]. Then, glucose is converted into ethanol and carbon dioxide with other enzymes related to Embden–Meyerhof–Panas (EMP). Thus, yeast uses sugar to grow and produce ethanol at the same time (Figure 2A). It can be seen that both of the TA and TVA contents increased slightly during fermentation (Figure 2B). An increase in the acid content of wine during the fermentation period resulted in suitable conditions for yeast growth [36].

Table 4 summarizes the distribution of the ethanol, TSS, TA, and TVA reference values in the samples for calibration and test sets. The content ranges of all chemical reference values in the samples for the calibration set covered those ranges found in the samples for the test set. Consequently, the variability of sample data in both calibration and prediction sets was considered appropriate for developing reliable NIR calibration models for ethanol, TSS, TA, and TVA predictions.

### 3.2. NIR Spectra of Pineapple Wines from the Fermentation Process

**Figure 3:**
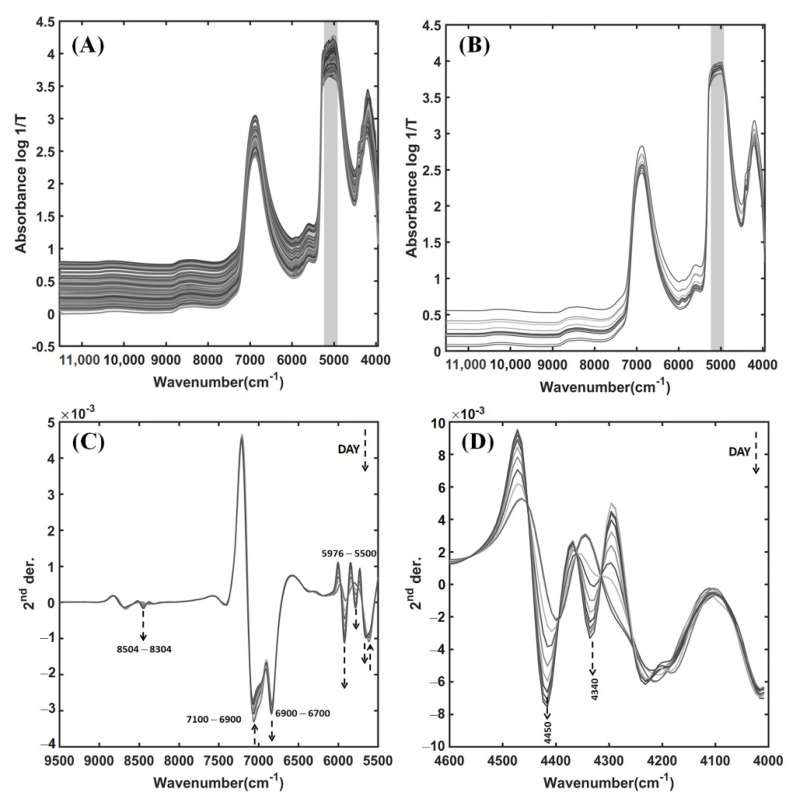
A total of 198 original NIR spectra in the 11,536–3952 cm^−1^ region of the pineapple wines during the fermentation process (**A**), 11 of the mean spectra of the fermentation samples from 0 to 10 days in the whole spectral region (**B**), and after performing second derivatives (SD) in the 9500–5500 (**C**) and 4600–4000 cm^−1^ spectral regions (**D**).

One hundred and ninety-eight of the original NIR spectra in the 11,536–3952 cm^−1^ region of pineapple wine samples obtained during fermentation using a liquid probe, and the eleven averaged spectra of the fermentation samples from 0 to 10 days in the whole spectral region, are shown in Figure 3A,B, respectively. A major component of pineapple wine is water. Therefore, a strong absorption band near 6900 and a saturated feature around 5000 cm^−1^ are assigned to the combination of OH symmetric and antisymmetric stretching modes, and the combination mode of the OH stretching and bending vibrations of water, respectively [15,37]. It is noted in Figure 3A,B that the saturated spectral region in a grey bar is excluded for model development. However, the spectral changes of the samples during different days of fermentation were not clearly visible in the original NIR spectra. Thus, the second derivatives (SD) were applied to reveal the significant NIR regions in the 11 averaged spectra of the fermentation samples from 0 to 10 days. Figure 3C,D presents the SD pretreated spectra in the 9500–5500 cm^−1^ range of pineapple wines for different fermentation dates. In Figure 3C, the SD pretreated spectra reveal the changes in NIR absorption bands around 8400, 6800, 5900, 5750, and 5650 cm^−1^ increased with fermentation time. Moreover, two dominant absorption bands can be seen near 4450 and 4340 cm^−1^ in the SD pretreated spectra of 4600–4000 cm^−1^ region that changed by increasing the fermentation time (Figure 3D). The absorption bands from ethanol production during the wine fermentation were previously reported in the 6060–5715 and 4545–4350 cm^−1^ spectral regions [13,38,39]. The former was due to the C–H stretch first overtones of the CH_3_ and CH_2_ groups, and the latter was assigned to the C–H stretch and C–H deformation combination from the CH_3_ group of ethanol [13,38,39]. The changes in the characteristic absorption bands observed in this study are similar to those reported by others. Therefore, they are related to characteristic bands for ethanol production from wine fermentation.

There are absorption bands around 7056 and 5610 cm^−1^ that decreased with fermentation time (Figure 3C). They were assigned to the O–H stretch first overtone and C–H stretch first overtone, respectively, which are characteristic bands for sugars [40,41]. The sugar contents are expressed by means of the TSS value. It is because the sugar contents are the highest among soluble solids dissolved in a pineapple wine sample. The characteristic bands for sugars decrease with fermentation time, corresponding to the process by which yeast converts sugars to ethanol. Furthermore, the functional groups of sugars and starch for the O–H stretch first overtone (6500 to 6300 cm^−1^), C–H stretch first overtone (5903 to 5650 cm^−1^), O–H stretch and C–O stretch combinations, and C–H combinations of stretch and deformation (4504 to 4250 cm^−1^) are expected to appear in the NIR spectra of pineapple wine (Table 5) [40,41]. In Figure 3C,D, the characteristic of changes involving such expected bands are found to increase in absorption over the time of fermentation. Although the sugar contents should be greatly reduced by yeast for ethanol production in pineapple winemaking, the contents of glucose and fructose are also increased by the enzyme invertase found in the growth phase of the yeast [35]. Therefore, the NIR spectra may convey two opposite directions of sugar changes due to the fermentation pathway by yeast.

Acidity in wine is expressed as the concentration of acids present, namely citric acid (TA) and acetic acid (TVA). From the literature, the chemical structures of both acids for the C–H stretch first overtone, C–H stretch second overtone, and C–H stretch and C=O stretch combinations are expected to appear in the spectral regions of 8504 to 8304, 5952 to 5600, and 4504 to 4200 cm^−1^, respectively [40,41]. Although the TA and TVA values increased over the fermentation time as shown in Figure 2B, the NIR bands involve the functional groups of the major constituents in pineapple wine, i.e., water, ethanol, and sugars, which also appeared around these areas as well. The major constituents in wine exhibited the dominant NIR bands where there may be overlap with the acid bands. It is because pineapple wine in the fermentation process has very low concentrations of citric acid (<0.5%) and acetic acid (<0.002%) (Table 2). Hence, the individual spectral regions associated with the citric and acetic acids cannot be clearly identified in the original and SD pretreated NIR spectra of pineapple wines. The NIR band assignments from the SD pretreated spectra of pineapple wine during the fermentation process are summarized in Table 5.

### 3.3. SCMWPLS Analysis

**Figure 4:**
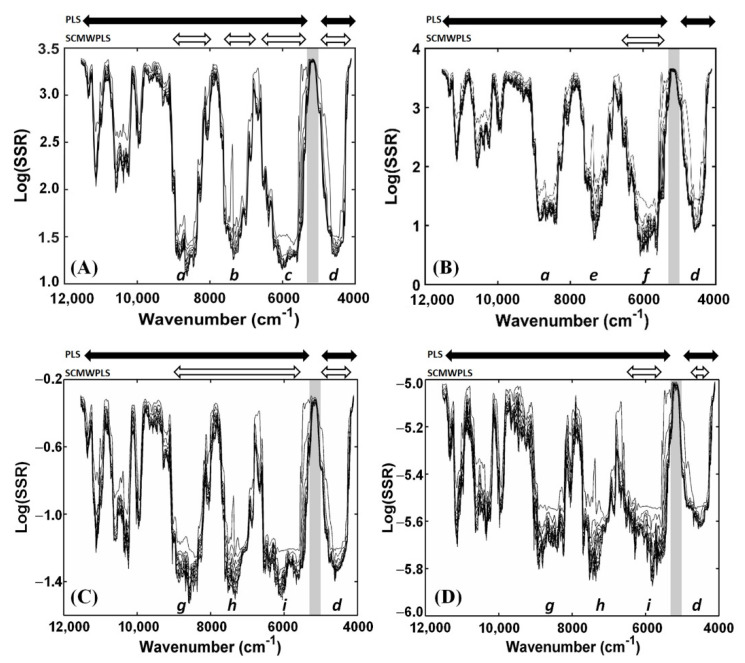
Residue lines for ethanol (**A**), total soluble solids (**B**), total acidity (**C**), and total volatile acids (**D**), obtained by MWPLSR using second derivative spectral data.

The original NIR data after performing the SD method were employed in the MWPLSR calculations for searching for the informative spectral regions for ethanol, TSS, TA, and TVA in pineapple wine spectra. The residue lines for ethanol, TSS, TA, and TVA obtained by MWPLSR for the whole NIR spectral region of 11,536–3952 cm^−1^ are shown in Figure 4A–D, respectively. In residual line spectra, each line represents a certain number of LVs. The top line shows the log(SSR) values of the first LV model, and then the LV model increases accordingly in the following lines. In this study, the maximum LVs number was set to 10 LVs, resulting in a total of 10 lines of the residual spectra. It is noted in Figure 4 that the saturated spectral region in a grey bar is excluded for model development.

Figure 4A shows the four obtained informative spectral regions of 9200–8000 (*a*), 7800–6800 (*b*), 6720–5256 (*c*), and 4976–4008 (*d*) cm^−1^ for ethanol calculated by MWPLSR. They correspond to the second (*a*) and first (*b*, *c*) overtones, and combination bands (*d*) from the functional groups of ethanol, respectively (Table 5). These informative spectral regions discovered by MWPLSR can easily be seen to encompass those bands assigned for ethanol from the SD pretreated spectra of pineapple wine samples (Figure 3C,D).

Four informative spectral regions of 9200–8000 (*a*), 7800–6904 (*e*), 6848–5256 (*f*), 4976–4008(*d*) cm^−1^ for TSS found by MWPLSR, are shown in Figure 4B. All informative spectral regions of *a*, *e*, *f*, and *d* for TSS cover the band assignments for sugars and related compounds given in Table 5. In Figure 4A,B, the third overtone bands for the 11,536 to 9800 cm^−1^ spectral region for ethanol and TSS show obviously high residue values (approximately > 2.3) from the residual spectral lines of two LVs. This line is the starting point for the suitability of the model dimensions built in this region, i.e., the fitness of residual lines is considering the line, showing the residue values decrease insignificantly as the number of LVs increases. Therefore, this third overtone spectral region was omitted in the optimization by SCMWPLS due to less spectral information of ethanol and TSS for the model developments.

Figure 4C,D presents the same for four informative spectral regions of 9400–7904 (*g*), 7896–6808 (*h*), 6800–5256 (*i*), and 4976–4008 (*d*) cm^−1^ for TA and TVA obtained by MWPLSR. The NIR band assignments that fall in these four informative spectral regions found by MWPLSR are described in Table 5. Although the individual spectral regions associated with both acids cannot be identified in the original and SD pretreated NIR spectra of pineapple wines, MWPLSR can suggest using the informative spectral regions of *g*, *h*, *i*, and *d* for both acids with the low SSR values. The sharp peaks around 11,536–9800 cm^−1^ of the residual line spectra for TA and TVA show the residue values at the last line (10 LVs) close to those values obtained from the four informative spectral regions (*g*, *h*, *i*, *d*). However, the residual lines can be fitted from two LVs in this region where they have higher SSR values than those given by the selected informative regions of *g*, *h*, *i*, and *d* (Figure 4C,D). Therefore, this spectral region of 11,536–9800 cm^−1^ is not chosen as the informative spectral region for TA and TVA. It is then excluded for optimization by SCMWPLS. For the informative spectral regions of ethanol, TSS, TA, and TVA obtained by MWPLSR, the SCMWPLS algorithm was performed to search for the optimized spectral regions.

### 3.4. Comparison of PLS Calibration Models

Statistical results for ethanol, TSS, TA, and TVA models developed by using the whole spectral region in both the original and SD pretreated NIR data and the optimized informative region obtained from SCMWPLS are compared in Table 6. In all cases, the spectral region from 5248 to 4984 cm^−1^, where the saturate water band is located, was removed.

The acceptable NIR models should show high *R*^2^ and RPD values and low RMSEC and RMSEP values. In addition, the best model for each analyte could be evaluated after performing the validation method by using an external test set. Therefore, the model gives the lowest RMSEP and the highest RPD, and it is the better model. The interpretation of *R*^2^ and RPD values qualify a model as good for screening with an *R*^2^ of 0.66 to 0.81 or RPD > 3, good for quality control with an *R*^2^ of 0.83 to 0.90 or RPD > 5, and excellent for all analytical tasks with an *R*^2^ > 0.91; RPD > 8 [41].

It can be seen from Table 6 that the PLS calibration model for ethanol obtained using the whole spectral region yields the lowest predictability among the prediction results for ethanol. PLS prediction results for ethanol using the whole spectral region of the original NIR spectral data are an RMSEP of 0.466% and an RPD of 7.36 at 4 LVs, while the model base on the whole spectral region after performing SD pretreatment gives a better prediction model with the lower RMSEP of 0.406%, a higher RPD of 8.44 at four LVs. Moreover, the SCMWPLS coupled with the SD pretreatment provides the optimized combination of 9104–7984, 7752–6704, 6600–5256, and 4976–4008 cm^−1^ regions. This optimized combination provides very good prediction results with the lowest RMSEP of 0.393%, the highest RPD of 8.72, and a high *R*^2^ of 0.984 with three LVs. These results are reasonably better than those calculated by using the whole spectral regions.

For TSS, the PLS prediction results of using the whole spectral region of the original NIR spectral data are an RMSEP of 0.441% and an RPD of 10.47 at five LVs, while the whole spectral region after performing SD pretreatment shows significant improvements with a lower RMSEP of 0.219% and a higher RPD of 21.08 at two LVs. In total, the MWPLSR suggested four individual informative spectral regions for TSS in the SD pretreated NIR spectra (Figure 4B). After performing SCMWPLS for these four informative regions, one spectral region of 6800–5360 cm^−1^ that provided the best prediction results, with the lowest RMSEP of 0.166 °Brix and the highest RPD of 27.82 with two LVs, was revealed. SCMWPLS improves the RMSEP and RPD values significantly, and the number of LVs is clearly reduced.

By comparison of the results listed in Table 6, one can find that the predictive performance of models for TA and TVA are lower than those models for ethanol and TSS predictions. The quality of models for TA and TVA can be classified as good for quality control (*R*^2^ > 0.88; averaged RPD = 3.07) and good for screening (*R*^2^ > 0.75 averaged RPD = 2.69), respectively. This was caused by the low concentrations of citric acid (TA) and acetic acid (TVA) in pineapple wine from the fermentation process. In addition, the concentration range and standard deviation of both acids are narrow, with 0.2880–0.4757% and 0.0514 of the SD for citric acid (TA), and 0.0011–0.0019% and 0.0002 of the SD for acetic acid (TVA). However, the best result for the calibration model of TA is obtained from the optimized combination of 9200–5408 and 4976–4008 cm^−1^ regions generated by SCMWPLS. It achieves improvement with the lowest RMSEP of 0.0181% and the highest RPD of 3.17 at two LVs. As for TVA, the optimized combination generated by SCMWPLS is composed of the 6504–5280 and 4504–4248 cm^−1^ regions. The optimized combination provides the best prediction result with the lowest RMSEP of 0.000105% and the highest RPD of 2.86 with two LVs.

One can see in Table 6 that the best models obtained by SCMWPLS could reduce the NIR spectral data points for model development. The smallest NIR spectral data were 181 points for building the TSS calibration model and the highest NIR spectral data were 597 points for the modelling of TA. The simultaneous monitoring of all four chemical changes could be performed by setting the spectral acquisition for the FT-NIR spectrometer to 616 spectral points (9200–5256 and 4976–4008 cm^−1^), in which these wavenumber variables cover the optimized region for all constituents found by SCMWPLS. Then, the measurement time will become faster than collecting the NIR spectral data for the whole region (915 spectral points).

**Figure 5:**
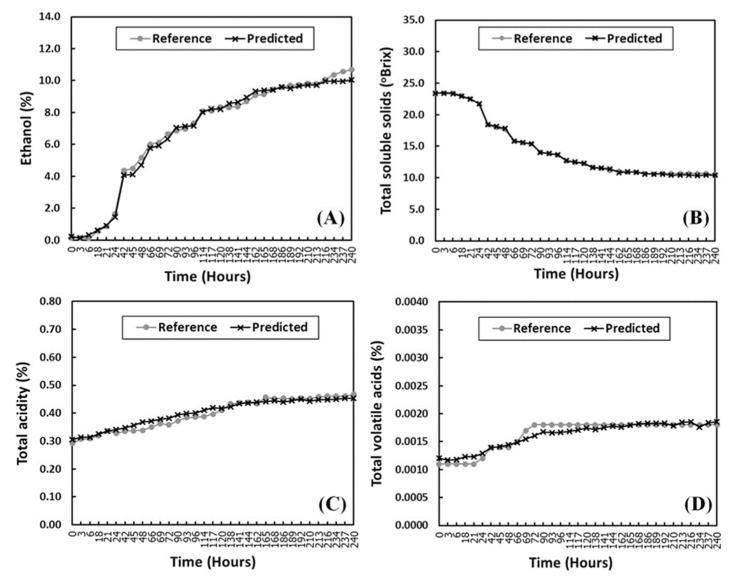
Comparison of quantitative analysis results for ethanol (**A**), TSS (**B**), TA (**C**), and TVA (**D**) in pineapple wine following the fermentation time by the best NIR models using SCMWPLS and the reference methods.

Figure 5 shows the NIR predicted and reference values of the independent test set versus the fermentation time using the best NIR models for ethanol, TSS, TA, and TVA obtained from SCMWPLS. The best predictive result is obtained from the calibration model for TSS prediction, where the NIR-predicted values did not differ from the references detected by a conventional method. This can be seen from the cross symbol showing the NIR prediction values overlaid with the circle symbol showing the reference values (Figure 5B).

The NIR prediction results calculated by the best models for ethanol prediction built by the use of optimized spectral region found by SCMWPLS also yield accurate results. However, a distinct difference between the reference values and the NIR prediction occurs during 234 to 240 h of fermentation (Figure 5A). It was almost the end of the fermentation process at this time, in which the ethanol production should be nearly constant. The NIR prediction value seems to show a more realistic change than the reference at this point. For TA and TVA, the prediction results obtained from the best models showed lower accuracy than the TSS and ethanol prediction results. Figure 5C,D shows the apparent difference between the reference and NIR predictive values of TA and TVA that occurred approximately from 48 to 114 h and around 18 and 69 to 96 h of fermentation, respectively. The reason may be that this period shows a high rate of ethanol production. As can be seen in Figure 5A, ethanol content gradually increases after 18 h and then increases rapidly from 24 to 114 h. Conversely, the TSS values show a corresponding decrease at the same time (Figure 5B). Therefore, both ethanol and CO_2_ are rapidly abundant in the fermentation sample. They can interfere with the observed NIR information due to the citric and acetic acids, and this may result in low accuracy for TA and TVA predictions at this period.

To assure the predictive performance of the best NIR models built by the optimized region from SCMWPLS, the bias confidence limits (*T_b_*) and the unexplained error confidence limits (*T*_UE_) were also employed as an indicator of NIR predictions in this study. The validation process through an independent test set provided the SEP and bias values, which should be compared with the calculated *T*_UE_ and *T_b_* values, respectively. When both the SEP and bias values were below these two confidence limits (SEP < *T*_UE_; bias < ±*T_b_*), this NIR model is considered to be accepted for its performance. The statistical results for the performance evaluation of the best models are summarized in Table 7. There is no doubt regarding an accurate predictive performance for ethanol and TSS models as the results show above. However, this statistical analysis was specially employed because the efficiency of the best models for TA and TVA should be taken into account. From Table 7, it can be seen that the statistical results obtained from the best models for TA and TVA also met the criteria. The interpretations of this result are that the SEP value is low enough to make it practically acceptable when it was lower than the calculated *T*_UE_ value, and the bias value was not significantly different from zero when it was lower than that calculated ±*T_b_*.

## 4. Conclusions

The results of present study demonstrated the potential of NIR spectroscopy coupling with SCMWPLS to enhance the predictive performance of NIR calibration models for simultaneously monitoring the changes in ethanol, total soluble solids, total acidity, and total volatile acids in pineapple fruit wine during the fermentation process. SCMWPLS could select and optimize informative spectral regions from the second derivative spectra of very complicated mixtures such as wine obtained by the FT-NIR fibre optic probe. The optimized informative regions are the combination of 9104–7984, 7752–6704, 6600–5256, and 4976–4008 cm^−1^ regions for ethanol, the 6800–5360 cm^−1^ region for TSS, the combination of 9200–5408 and 4976–4008 cm^−1^ regions for TA, and the combination of 6504–5280 and 4504–4248 cm^−1^ regions for TVA. The quality of their PLS calibrations is improved in comparison with those obtained using the whole region. Furthermore, the present study has verified the advantages of the NIR liquid probe in combination with SCMWPLS for direct NIR measurements in pineapple wines from the fermentation process without sample preparation. Therefore, the best models obtained from these tools provided good prediction results with acceptable statistics and especially the use of a small number of spectral data points that will make faster NIR measurement possible. However, further cases or device designs for liquid probe measurement should be considered to protect the probe from being disturbed by the CO_2_ and microparticles (if the interference has a particle size smaller than the probe slit < 1 mm) found in the fermentation system in order to stabilize the NIR signal and improve the prediction of low-concentration constituents.

## Figures and Tables

**Table 1:** Literature reviews of the applications of NIR or VIS–NIR spectroscopy for quantitative analysis of constituents in wine during the fermentation process.

Sample	VIS-NIR or NIR Range (cm^−1^)	Measurement Mode	Sample Preparation	Chemometric Method	Analyst	RMSEP or ^a^ RMSECV or ^b^ SEP or ^c^ SECV
Apple wine [20]	12,000–4000	Transmission; Quartz cuvette	Centrifugation and filtration	PLS	Soluble solid content	0.60%
					pH	0.08
					Total acidity	0.02 g 100 mL^−1^
					Total ester content	0.10 g L^−1^
Apple wine [21]	12,000–4000	Transflection; Liquid probe	Centrifugation	PLS	Alcohol strength	4.25 mL L^−1^
					Titratable acidity	0.21 g L^−1^
Jujube wine [22]	10,526–6060	Transmission; Quartz cuvette	Filtration	PLS	Alcohol	^a^ 0.70%
Red wine [13]	25,000–4000	Transmission; Quartz cuvette	Centrifugation	PLS	Malvidin-3-glucoside	^c^ 17.50–31.50 mg L^−1^
					Pigmented polymers	^c^ 3.20–26.80 mg L^−1^
					Tannins	^c^ 49.10–131.20 mg L^−1^
Rice wine [19]	10,000–4000	Transmission; Quartz cuvette	Centrifugation	RCA–SVM–PLS	Ethanol	2.60 g L^−1^
				GA–SVM–PLS	Total acid	0.10 g L^−1^
White wine [23]	14,285–9434	Transmission; Quartz cuvette	Filtration	PLS	Volumic mass	^a^ 4.18 g (dm^3^)^−1^
					Reducing sugars	^a^ 10.35 g L^−1^

RMSEP = Root mean square error of prediction; ^a^ RMSECV = Root mean square error of cross validation; ^b^ SEP = Standard error of prediction; ^c^ SECV = Standard error of cross validation; RCA = Regression coefficient analysis; SVM = Support vector machine; GA = Genetic algorithm.

**Table 2:** Summary of statistical computations used to estimate NIR model performance.

Statistical Terms	Computations
Coefficient of determination (*R*^2^)	$R^{2}=\frac{{\left(\sum_{i=1}^{n}\left(x_{i}-\bar{x}\right)\left(y_{i}-\bar{y}\right)\right)}^{2}}{\sum_{i=1}^{n}{\left(x_{i}-\bar{x}\right)}^{2}\sum_{i=1}^{n}{\left(y_{i}-\bar{y}\right)}^{2}}$
Root mean square error (RMSE) RMSEC in the calibration set RMSEP in the test set	$\mathrm{RMSE}=\sqrt{\frac{1}{n}\overset{n}{\underset{i=1}{\sum }}{\left(x_{i}-y_{i}\right)}^{2}}$
Standard error of prediction (SEP)	$\mathrm{SEP}=\sqrt{\frac{1}{\left(n-1\right)}\overset{n}{\underset{i=1}{\sum }}{\left(x_{i}-y_{i}-\mathrm{Bias}\right)}^{2}}$
Bias	$\mathrm{Bias}=\frac{1}{n}\overset{n}{\underset{i=1}{\sum }}\left(x_{i}-y_{i}\right)$
Residual predictive deviation (RPD)	$\mathrm{RPD}=\frac{\mathrm{SD}}{\mathrm{RMSEP}}$
Bias confidence limits (*T_b_*) =	$T_{b}=\pm \frac{t_{\left(1-\alpha /2\right)}\;\times \;\mathrm{SEP}}{\sqrt{n}}$
Unexplained error confidence limits (*T*_UE_)	$T_{\mathrm{UE}}=SEC\times \sqrt{F_{\left(\alpha ,\;\nu ,\;Μ\right)}}$

$x_{i}$ = the reference value of sample *i*; $\bar{x}$ = the average of reference values of samples; $y_{i}$ = the predicted value of sample *i*; $\bar{y}$ = the average of predicted values of samples; *n* = number of samples; SD = the standard deviation of reference values; $t_{\left(1-\alpha /2\right)}$ = the *t*-value for a two-tailed *t*-test with degrees of freedom associated with *SEP* (type I error); α = the significance level of 0.05; *F* = the *F*-value for *F*-test with degrees of freedom associated with SEP ($\nu =n_{p}-1$) and SEC $\left(Μ=n_{c}-LVs-1\right)$.

**Table 3:** The average content of ethanol, total soluble solids, total acidity, and total volatile acids in the samples from two batches of the pineapple wine fermentation process.

Fermentation Day	Ethanol (%)	Total Soluble Solids (°Brix)	Total Acidity (%)	Total Volatile Acids (%)
0	0.06	23.70	0.29	1.30 × 10^−3^
1	0.40	23.17	0.32	1.30 × 10^−3^
2	3.91	18.42	0.35	1.28 × 10^−3^
3	5.54	15.88	0.38	1.50 × 10^−3^
4	6.74	13.87	0.39	1.67 × 10^−3^
5	7.75	12.33	0.41	1.65 × 10^−3^
6	8.49	11.35	0.42	1.75 × 10^−3^
7	9.06	10.83	0.44	1.80 × 10^−3^
8	9.71	10.52	0.44	1.78 × 10^−3^
9	10.08	10.33	0.45	1.78 × 10^−3^
10	10.76	10.25	0.46	1.80 × 10^−3^

**Table 4:** Content distribution of ethanol, total soluble solids, total acidity, and total volatile acids in the calibration set (*n* = 198) and test set (*n* = 99) determined by the reference methods.

Parameters	Sample Set	Minimum	Mean	Maximum	Standard Deviation
Ethanol (%)	Calibration	0.04	6.61	11.56	3.60
	Test	0.12	6.83	10.68	3.43
Total soluble solids (°Brix)	Calibration	10.00	14.57	24.20	4.88
	Test	10.53	14.55	23.50	4.62
Total acidity (%)	Calibration	0.29	0.40	0.48	0.05
	Test	0.29	0.40	0.47	0.06
Total volatile acids (%)	Calibration	1.10 × 10^−3^	1.60 × 10^−3^	1.90 × 10^−3^	2.00 × 10^−4^
	Test	1.10 × 10^−3^	1.60 × 10^−3^	1.80 × 10^−3^	3.00 × 10^−4^

**Table 5:** The band assignments of significant NIR regions with absorption changes during pineapple wine fermentation from the second derivative pretreated spectra *^a^*.

Wavenumber (cm^−1^)	Band Assignment	Substance [40,41]
8900–8504	O–H	Water [37]
8504–8304 *^b^*	C–H stretch 2nd overtones of –CH_3_, –CH_2_	Ethanol, Sugars, Citric acid, Acetic acid
7100–6900 *^b^*	O–H stretch 1st overtones	Sugars
6900–6700 *^b^*	O–H stretch 1st overtones	Ethanol (primary alcohols), Starch
6896 *^b^*	C=O stretch 1st overtones from carbonyl group	Citric acid, Acetic acid
6850	O–H	Water [36]
6500–6300	O–H stretch 1st overtones	Starch
5976–5500 *^b^*	C–H stretch 1st overtones of –CH_3_, –CH_2_	Ethanol [13,38,39,42], Sugars, Citric acid, Acetic acid
4504–4250 *^b^*	C–H combinations of stretch and deformation from the CH_3_ group	Ethanol [13,38,39,42,43]
4504–4250 *^b^*	O–H stretch and C–O stretch combinations, C–H combinations of stretching and deformation	Sugars [43], Glucose, Cellulose, Starch
4504–4250 *^b^*	C–H stretch and C=O stretch combinations	Citric acid, Acetic acid

*^a^* = The spectral regions of 9500–5500 and 4600–4000 cm^−1^; *^b^* = The intensity changes according to the fermentation date; [40,41] = All substances in Table 5 are referred to in reference numbers 40 and 41; Additional references to some substances are annotated by superscript as reference numbers.

**Table 6:** Statistics results for PLS calibration models of ethanol, TSS, TA, and TVA contents for pineapple wine in fermentation developed using uncorrected spectrum or second derivative corrected spectrum in the whole regions and those regions selected by SCMWPLS.

Parameters	Methods	Preprocessing	LVs	*R* ^2^	RMSEC	RMSEP	RPD	Spectral Data Points
Ethanol (%)	PLS	none	4	0.973	0.588	0.466	7.36	915
	PLS	SD	4	0.985	0.438	0.406	8.44	901
	SCMWPLS (cm^−1^) 9104–7984, 7752–6704, 6600–5256, 4976–4008	SD	3	0.984	0.457	0.393	8.72	564
TSS (°Brix)	PLS	none	5	0.997	0.269	0.441	10.47	915
	PLS	SD	2	0.995	0.330	0.219	21.08	901
	SCMWPLS (cm^−1^) 6800–5360	SD	2	0.996	0.286	0.166	27.82	181
TA (%)	PLS	none	2	0.883	0.174 × 10^−1^	0.182 × 10^−1^	3.15	915
	PLS	SD	2	0.892	0.167 × 10^−1^	0.199 × 10^−1^	2.88	901
	SCMWPLS (cm^−1^) 9200–5408, 4976–4008	SD	2	0.894	0.166 × 10^−1^	0.181 × 10^−1^	3.17	597
TVA (%)	PLS	none	6	0.776	0.112 × 10^−3^	0.117 × 10^−3^	2.56	915
	PLS	SD	5	0.753	0.118 × 10^−3^	0.113 × 10^−3^	2.65	901
	SCMWPLS (cm^−1^) 6504–5280, 4504–4248	SD	6	0.761	0.116 × 10^−3^	0.105 × 10^−3^	2.86	187

Whole region for PLS = 11536–5256, 4976–3952 cm^−1^; SD = second derivatives; LVs = number of latent variables; *R*^2^ = coefficient of determination; RMSEC = root mean squares error of calibration; RMSEP = root mean squares error of prediction; RPD = Residual predictive deviation.

**Table 7:** Statistics for assessment of the model performance.

NIR Models by SCMWPLS	Statistics	Obtained Results	Criterion	Performance
Ethanol (%)	SEP	0.374	*T*_UE_ = 0.554	accepted
	Bias	0.128	*T_b_* = ±0.132	accepted
TSS (°Brix)	SEP	0.164	*T*_UE_ = 0.346	accepted
	Bias	0.029	*T_b_* = ±0.058	accepted
TA (%)	SEP	0.175 × 10^−1^	*T*_UE_ = 0.201 × 10^−1^	accepted
	Bias	0.005	*T_b_* = ±0.006	accepted
TVA (%)	SEP	0.105 × 10^−3^	*T*_UE_ = 0.140 × 10^−3^	accepted
	Bias	0.012 × 10^−3^	*T_b_* = ± 0.037 × 10^−3^	accepted

*T*_UE_ = unexplained error confidence limits (*α* = 0.05); *T_b_* = bias confidence limits (*α* = 0.05).

## Data Availability

The data presented in this study are available in the article.
